# May-Thurner Syndrome and Its Possible Influence on Pulmonary Embolism Severity: A Case Report

**DOI:** 10.7759/cureus.105017

**Published:** 2026-03-11

**Authors:** Elias Nabhan, Edouard Naoum Nehme, Alexandrina-Paula Vana, Nabil Poulos

**Affiliations:** 1 Cardiovascular Medicine, Centre Hospitalier Josephine Baker, Gonesse, FRA

**Keywords:** deep vein thrombosis, embolization dynamics, iliac vein compression, may-thurner syndrome, pulmonary embolism

## Abstract

The management of recurrent venous thromboembolism requires a high index of suspicion for underlying structural anomalies such as May-Thurner syndrome (MTS). Although the prothrombotic consequences of venous compression are well established, its influence on embolization dynamics has not been clearly defined. We report the case of a 38-year-old female with recurrent left-sided iliofemoral deep vein thrombosis (DVT) who presented with progressive calf pain without cardiopulmonary symptoms. Venous Doppler ultrasound demonstrated extensive thrombosis of the iliac, femoral, popliteal, and tibial veins. Pulmonary computed tomography angiography (CTA) revealed a clinically silent distal pulmonary embolism (PE). Abdominal CTA, using a triple-phase acquisition protocol, showed an 80% diameter stenosis of the left common iliac vein due to compression by the right common iliac artery, consistent with MTS. Thrombophilia testing was negative. The patient was treated successfully with oral anticoagulation and graduated compression stockings (GCS) as she declined endovascular stenting. This case confirms the established association between MTS and recurrent left-sided thrombosis. It also raises the theoretical possibility that severe iliac vein stenosis could modulate embolus size through altered venous flow dynamics. However, this hypothesis remains speculative and requires confirmation in mechanistic and epidemiologic studies.

## Introduction

Venous thromboembolism (VTE), including deep vein thrombosis (DVT) and pulmonary embolism (PE), is a major cause of cardiovascular morbidity and mortality worldwide [1]. PE occurs when a thrombus migrates from the deep venous system into the pulmonary arterial system, resulting in a range of clinical presentations from no symptoms to obstructive shock [2].

In addition to the well-documented classical risk factors for VTE, including recent surgery, immobility, cancer, pregnancy, and hormonal therapy, structural venous abnormalities contribute to thrombogenesis [3]. May-Thurner syndrome (MTS), also known as Cockett syndrome, results from chronic compression of the left common iliac vein by the right common iliac artery against the lumbar vertebral body [4]. This compression injury occurs as a result of the pulsatile arterial forces, leading to injury of the endothelium, hyperplasia of the intima, and development of fibrous spurs within the lumen of the left common iliac vein, which leads to venous stasis and thrombosis [4,5].

Imaging studies have shown that iliac vein compression is relatively common in the general population; in a CT-based study of asymptomatic individuals, approximately 24% demonstrated at least 50% compression of the left common iliac vein [6]. While the thrombogenic role of MTS is well documented, its potential impact on embolization dynamics has not been systematically investigated. We describe a case that emphasizes the association between MTS and recurrent DVT and raises the question of a theoretical hemodynamic mechanism that may influence embolic burden.

## Case presentation

A 38-year-old female presented with a 10-day history of progressive left calf pain. She reported chronic left leg edema without functional impairment, exacerbated by prolonged standing. At age 30, she experienced an extensive uninvestigated left-sided iliac and femoral DVT that resolved after treatment with oral anticoagulation for three months.

The patient had a normal body mass index (BMI). She denied smoking, alcohol consumption, oral contraceptive use, or other hormonal therapy. She denied any history of recent surgery, trauma, immobilization, or long-distance travel. She was not taking any medication.

Vital signs were stable. Oxygen saturation was 98% on room air. Cardiopulmonary examination was normal. Physical examination revealed unilateral swelling of the left lower limb with calf tenderness and a mild increase in calf circumference compared with the contralateral side, without erythema, skin discoloration, or signs of chronic venous insufficiency. Peripheral arterial pulses were palpable and symmetric.

**Figure 1:**
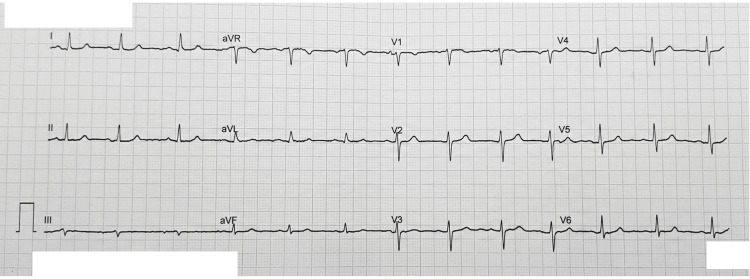
Electrocardiogram (ECG) of the patient at presentation.

The electrocardiogram (ECG) showed a sinus rhythm without right ventricular strain (Figure 1).

**Figure 2:**
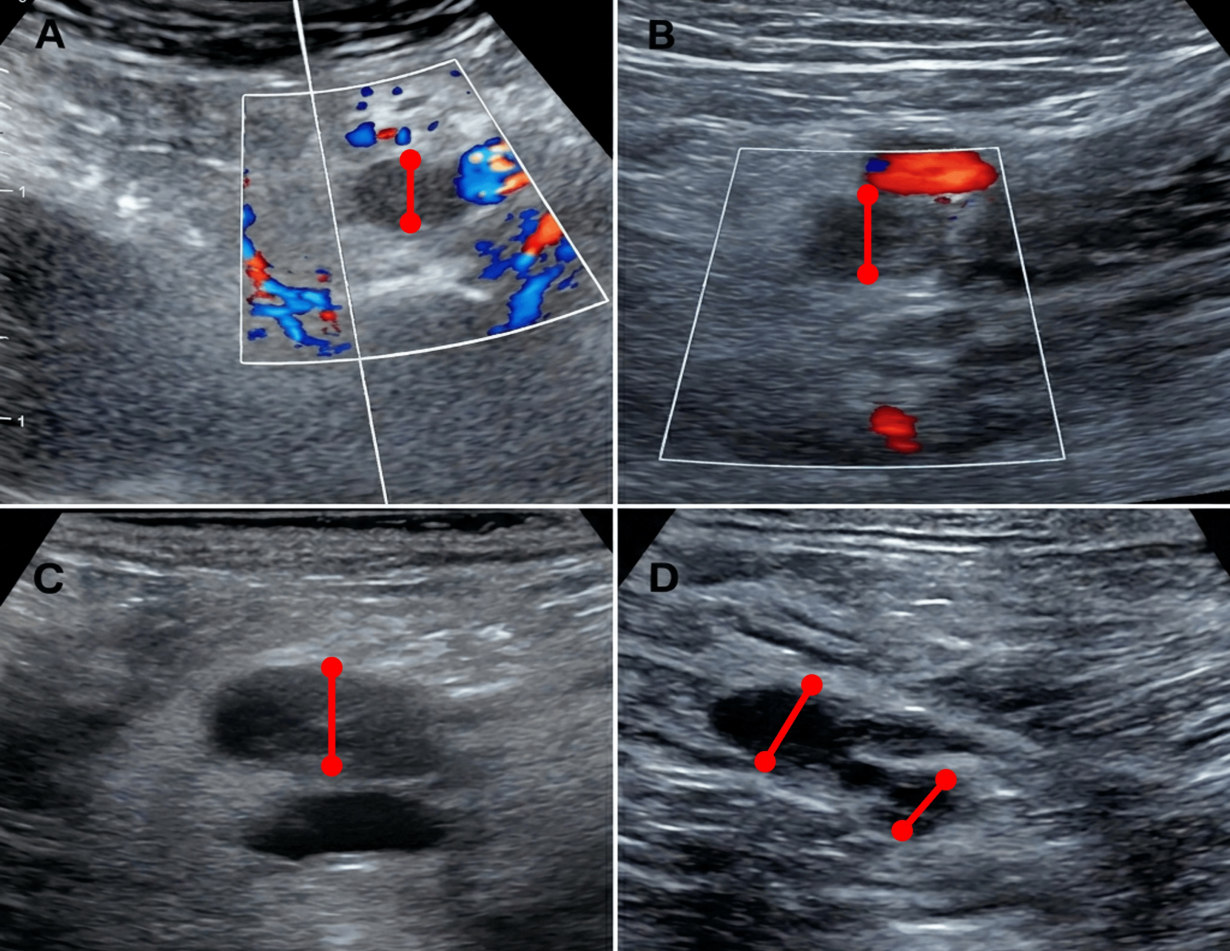
Venous Doppler ultrasound demonstrating extensive thrombosis of the left lower limb. The measurements represent the maximal venous diameter in the affected non-compressible segments: left external iliac vein (15 mm; A), common femoral vein (12 mm; B), popliteal vein (11 mm; C), and posterior tibial veins (7 mm on the left and 5 mm on the right; D).

Transthoracic echocardiography demonstrated normal biventricular size and function with a left ventricular ejection fraction of 60% and no signs of pulmonary hypertension. Lower-limb venous doppler ultrasound demonstrated an acute non-compressible thrombus with absent color doppler flow involving the left external iliac, common femoral, popliteal, and posterior tibial veins, consistent with extensive proximal DVT (Figure 2).

Given the proximal extension of the thrombus, pulmonary CTA was performed and revealed an asymptomatic subsegmental PE in the right lower lobe without right ventricular enlargement or cardiac biomarker elevation. Laboratory investigations are summarized in Table 1. Thrombophilia screening, performed before anticoagulation therapy, including factor V Leiden mutation, prothrombin gene mutation (G20210A), protein C deficiency, protein S deficiency, antithrombin III deficiency, and antiphospholipid antibodies, was negative.

**Table 1:** Laboratory values obtained at admission before anticoagulation therapy. NT-proBNP: N-terminal pro-B-type natriuretic peptide; β-hCG: β-human chorionic gonadotropin

Parameter	Result	Reference Range
Hemoglobin	13 g/dL	12-16 g/dL
White blood cells	8100 /µL	4000-10000 /µL
High-sensitivity troponin	<3 ng/L	<45 ng/L
NT-proBNP	<35 pg/mL	<125 pg/mL
C-reactive protein	3 mg/L	0-3 mg/L
D-dimers	1.7 µg/mL	<0.50 µg/mL
Creatinine	65 µmol/L	50-100 µmol/L
Homocysteine	9 µmol/L	3.2-10.7 µmol/L
Antithrombin III activity	100%	80-120 %
Protein C activity	104%	70-150 %
Total Protein S activity	80%	50-150 %
β-hCG	<2 IU/L	<5 IU/L
Factor V Leiden mutation (Arg506Gln)	Negative	Negative
Prothrombin gene mutation (G20210A)	Negative	Negative

**Figure 3:**
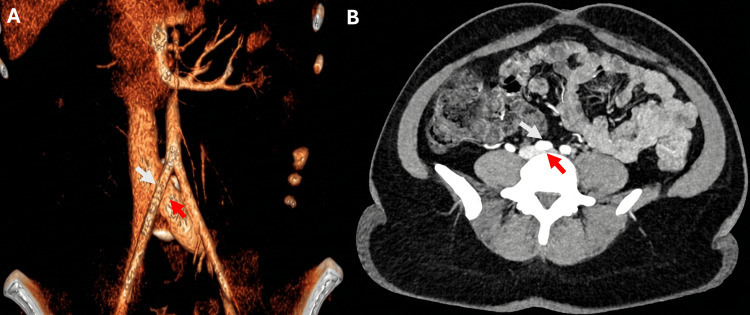
Abdominal computed tomography angiography showing compression of the left common iliac vein (red arrow) by the right common iliac artery (white arrow) on coronal (A) and axial (B) views.

Abdominal computed tomography angiography (CTA), using a triple-phase protocol, demonstrated an 80% diameter stenosis of the left common iliac vein with a minimal luminal diameter of 0.15 cm compared with 0.75 cm in the contralateral segment due to compression of the left common iliac vein by the right common iliac artery, consistent with MTS without significant collateral veins (Figure 3).

The patient was referred for vascular evaluation for possible endovascular intervention. After multidisciplinary discussion, she declined iliac vein stenting and opted instead for non-invasive management. Anticoagulation was initiated with rivaroxaban 15 mg twice daily for 21 days, followed by a lifelong maintenance dose of 20 mg once daily. She was also prescribed knee-high graduated compression stockings, class III (30-40 mmHg), for a 12-month duration. Repeated lower limb Doppler ultrasound showed a decreased size of the DVT at three months and complete resolution at six months. No clinical features of chronic venous insufficiency, such as persistent edema, skin hyperpigmentation, or venous claudication, were observed.

## Discussion

**Figure 4:**
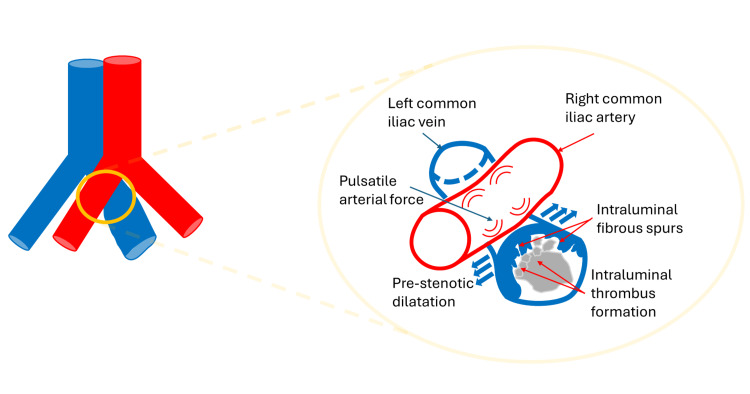
A schematic illustration of the proposed mechanism showing how chronic arterial pulsations induce the formation of intraluminal fibrous spurs, which act as a nidus for thrombus development. This figure was created by the authors using Microsoft PowerPoint (Microsoft Corporation, Redmond, WA).

MTS is a clinically significant but underdiagnosed cause of left-sided DVT [4,5]. It has been noted via imaging studies that patients with left-sided iliofemoral DVT present with a high incidence of underlying iliac vein compression [6]. The left common iliac vein is compressed between the overlying right common iliac artery, which results in venous irritation, endothelial injury, and progressive narrowing of the lumen, ultimately leading to the development of venous thrombi [4]. Histological studies have demonstrated the presence of intraluminal fibrous spurs in the compressed left common iliac vein, which result from chronic arterial pulsations and promote thrombosis through endothelial injury and venous stasis [4]. A schematic illustration of this mechanism is shown in Figure 4.

One possible explanation for the presence of a clinically silent distal PE despite extensive iliofemoral thrombosis in our case is that the iliac vein stenosis may have limited the passage of large thrombotic fragments into the inferior vena cava. In this context, the presence of iliac vein stenosis raises several questions regarding embolization dynamics; however, no protective effect can be inferred from a single case. Interestingly, similar observations have been suggested by Chan et al., who reported that significant left common iliac vein stenosis (>70% or luminal diameter <4 mm) was associated with lower odds of symptomatic PE, leading the authors to hypothesize that severe iliac narrowing may function as a mechanical barrier to large emboli [7].

In our case, axial abdominal CTA demonstrated severe focal narrowing of the left common iliac vein, with a minimal luminal diameter of 0.15 cm compared with 0.75 cm in the contralateral reference segment, corresponding to approximately 80% diameter stenosis. This critically reduced lumen measuring approximately 1.5 mm in diameter could possibly limit the passage of large, cohesive thrombus fragments originating distal to the compression site, promoting fragmentation prior to entry into the inferior vena cava. In this situation, emboli reaching the pulmonary circulation might preferentially be smaller and lodge distally rather than producing central or massive PE.

Nonetheless, the mechanism proposed here lacks any scientific validation. Stenotic segment shear forces/turbulence could also augment thrombus breakup and embolization probability. In addition, thrombus propagation proximal to the compressed segment could allow embolization without traversing a critically narrowed lumen. In addition, patients with chronic compression of the iliac vein can sometimes develop collateral pathways in the pelvic venous system [8]. These networks of veins, which include internal iliac veins, lumbar veins, and pelvic veins, may allow thrombus to reach the inferior vena cava, bypassing the stenotic area and the narrowed lumen [8,9]. The existence of these bypass veins may also complicate the dynamics of embolization and provide less certainty to the protective effect of an iliac stenosis. Therefore, the detection of a clinically silent PE in this patient despite extensive iliofemoral thrombosis should be interpreted cautiously. To date, there are no mechanistic or epidemiologic data demonstrating reduced incidence of massive PE among patients with MTS. Larger observational or hemodynamic studies would be required to determine whether iliac vein compression significantly influences embolic burden or severity. The treatment of MTS has changed considerably since advances in endovascular treatment strategies began to emerge. The current literature supports catheter-directed thrombolysis or thrombectomy, followed by iliac vein stenting, in patients with symptoms of MTS, and results show that those interventions produce good long-term outcomes and high vein patency rates, as well as decreased rates of post-thrombotic syndrome [10-12].

Anticoagulation alone treats the thrombotic event but does not correct the underlying mechanical compression; therefore, these patients are at high risk for developing recurrent thrombus [12]. As a result, there is an increasing trend toward performing endovascular stenting in patients with symptoms of MTS who also have significant venous outflow obstruction and/or have experienced recurrent venous thrombus, but optimal patient selection remains unclear [13]. Our patient declined invasive treatment and chose long-term anticoagulation therapy. Close follow-up remains essential to monitor for recurrent thrombosis and reconsider mechanical correction if symptoms recur.

This case report has several limitations. It describes a single clinical observation and is therefore unable to establish a causal relationship between iliac vein stenosis and PE severity. Additionally, the hypothesis that venous compression may affect embolus size is only speculative in nature and lacks mechanistic support. Finally, pelvic collateral venous pathways may bypass the stenotic segment, further limiting the plausibility of the proposed mechanism. Larger clinical studies and hemodynamic modelling would therefore be required to evaluate this hypothesis.

## Conclusions

MTS should be considered in patients presenting with recurrent or unexplained left-sided DVT, particularly in the absence of conventional risk factors. In this case, the coexistence of extensive iliofemoral thrombosis without documented collateral veins, along with a clinically silent distal PE, raises the theoretical possibility that iliac vein stenosis may influence embolization dynamics. While this observation suggests a possible modulatory role of venous compression on embolus size, such an effect remains hypothetical and unproven.
